# Mumps Vaccines: Current Challenges and Future Prospects

**DOI:** 10.3389/fmicb.2020.01999

**Published:** 2020-08-20

**Authors:** Iman Almansour

**Affiliations:** Department of Epidemic Diseases Research, Institute for Research and Medical Consultations (IRMC), Imam Abdulrahman Bin Faisal University, Dammam, Saudi Arabia

**Keywords:** mumps, vaccine, MMR, immunity, hemagglutinin-neuraminidase, vaccine efficacy, safety

## Abstract

Five decades have passed since the first mumps vaccine was licensed. Over this period, a resurgence of mumps infections has been recorded worldwide. Although global mumps infections have been controlled through vaccination, outbreaks are still on the rise, including in populations with high vaccination coverage. Several epidemiological studies suggest that this infectious virus continues to be a worldwide public health threat. The development and deployment of an improved, prophylactic mumps vaccine that provides long-lasting protection is indeed a priority. The purpose of this review is to provide an immuno-biological perspective on mumps vaccines. Here, we review the virology of mumps, licensed mumps vaccines, and the typical immune responses elicited following mumps vaccination. Furthermore, we discuss the limitations and challenges of the currently licensed mumps vaccines and provide strategies for the development of an improved mumps vaccine.

## Introduction

Mumps is a highly contagious viral infection first described by Hippocrates in the book *Epidemics* in the 5th century BC. The mumps virus was first isolated and cultured in 1945, and humans are its only known natural host. The mumps virus can be transmitted *via* respiratory droplets, fomites, or personal contact. The average incubation period from initial exposure to the onset of symptoms is 15–24 days, with a median onset of 19 days (Meyer, 1962; Richardson et al., 2001; Hviid et al., 2008). Once symptoms appear, a typical mumps infection is characterized by inflammation of the parotid glands. Some infected individuals develop severe complications such as orchitis, pancreatitis, septic meningitis, and deafness.

The first mumps vaccine was licensed in 1967. Soon after, the mumps vaccine was included as part of the trivalent measles, mumps, and rubella (MMR) vaccine. Today, the MMR vaccine is given in two doses as part of a routine immunization schedule in many countries. Although widespread use of the two-dose MMR vaccine largely reduced mumps incidence among school children by the 1990s, there has been a significant increase in the number of mumps outbreaks since. Additionally, vaccine efficacy and safety remains a concern. The re-emergence of mumps infections makes it worthwhile to review the virology of mumps, the immunity achieved after vaccination, and prospects to develop mumps vaccines with enhanced immunity in the future.

## Mumps Virology

The mumps virus is an enveloped, non-segmented, negative-sense RNA virus belonging to the *Rubulavirus* genus of the *Paramyxoviridae* family. When viewed using electron microscopy, the virions are spherical and pleomorphic in shape, ranging in size from 100 nm to 800 nm (McCarthy and Johnson, 1980). The mumps genome consists of 15,384 nucleotides encoding seven major proteins: fusion (F), hemagglutinin-neuraminidase (HN), nucleocapsid (N), large (L), matrix (M), phosphoprotein (P), and small hydrophobic (SH; Carbone and Rubin, 2007). The P gene encodes three transcripts, which are then translated into the P, V, and I proteins, respectively (Paterson and Lamb, 1990). The L and P proteins collectively form the RNA-dependent RNA polymerase, which acts as a replicase to copy negative-sense RNA into positive-sense RNA and also as a transcriptase to generate messenger RNAs (mRNAs; Kingston et al., 2004, 2008). The V protein blocks interferon (IFN)-β-induced signal transduction in host cells (Kubota, et al., 2001, 2005; Ulane et al., 2003). The N protein encapsulates the viral genome, forming the viral core, and the M protein links the viral core to the virus membrane in mature virions.

The HN protein facilitates viral attachment to host cells by binding to sialic acid receptors on the cell surface. Together, the HN and F proteins mediate virus-to-cell and cell-to-cell fusion, thereby enabling the virus to spread. Additionally, HN is the immunodominant antigen. Binding and neutralizing antibodies recovered from vaccinated individuals and convalescent patients are directed primarily against the HN glycoprotein and, to a lesser extent, against the F protein (Tanaka et al., 1992; Cusi et al., 2001; Matsubara et al., 2012).

The SH protein is also located on the viral membrane where blocks tumor necrosis factor-α, thus allowing the virus to evade the host’s antiviral response (Wilson et al., 2006). SH protein expression had no effect on virus replication *in vitro*; however, *in vivo* studies suggest the SH protein can play a role in viral pathogenesis (Takeuchi et al., 1996). SH has the most genetic diversity of all the genes in the mumps genome. Therefore, SH is used as the basis for mumps genotyping and epidemiological surveillance. Currently, 12 mumps genotypes have been identified (A through N, excluding E and M).

## Immunity to Mumps

Mumps immunity is generated primarily through antibodies targeting the viral HN protein. The SH protein is not known to be immunogenic, and antibodies against it are rarely recovered from infected or vaccinated individuals. The strongest immunoglobulin G (IgG) responses are directed against the N protein; however, NP antibodies are non-neutralizing. The protective efficacies of mumps antibodies following the first and second doses of the MMR vaccine are 78% (49–91%) and 88% (66–95%), respectively, making the mumps vaccine the least effective component of the trivalent MMR vaccine (CDC, 1974; Schaffzin et al., 2007; Marin et al., 2008; Marin, 2018).

Enzyme immunoassays are typically used to detect immunoglobulin M antibodies to confirm an immune response to mumps vaccination or infection. Mumps immunity is typically assessed by measuring neutralizing-antibody responses directed against mumps HN and F proteins. The plaque reduction neutralization (PRN) assay is the gold standard to assess immunoprotection. The PRN assay is used to determine the threshold concentration of neutralizing mumps antibodies necessary in a patient’s serum to reduce viral plaque formation in populations of cultured cells by 50% (Buynak et al., 1967; Christenson and Böttiger, 1990; Crowley and Afzal, 2002). The primary challenge for mumps vaccines is the lack of correlation between the immune response elicited from vaccination and level of protection against mumps infection conferred by the vaccine.

Limited data regarding cellular immunity to the mumps virus are available. Hyöty et al. (1986) demonstrated significant but temporary lymphocyte blast transformation in vaccinated individuals. In another study, the mumps vaccine provoked cell-mediated responses, as measured by the lymphocyte blast transformation test (Ilonen et al., 1984). Vaccination of infants at 6, 9, or 12 months of age resulted in mumps-specific T cell proliferation and IFN-γ production (Gans et al., 2001, 2003). A long-term study of the protective effects of mumps vaccination documented lymphoproliferative responses in two-thirds of vaccinated individuals 6 years after vaccination (Dhiman et al., 2005). Similarly, another study demonstrated persistent lymphoproliferative and IFN-γ effects after vaccination (Jokinen et al., 2007). Hanna-Wakim et al. (2008) reported 70% of vaccinated and 80% of infected individuals showed T cell immunity. In that study, T cell proliferation and IFN-γ, in addition to memory cells, were detected 10 years after vaccination or infection.

## Currently Licensed Mumps Vaccines

Although mumps can be administered as a monovalent vaccine, it is typically given as a part of the trivalent MMR vaccine or the tetravalent measles, mumps, rubella, and varicella (MMRV) vaccine. Commercially available MMR and MMRV vaccines are based on live attenuation of each of the constituent viruses, which can be administered as a single dose or double dose. Although, the MMR vaccine is given routinely in many countries around the world, the mumps strains used in the vaccine formulations vary widely among different countries (Table 1).

**Table 1:** List of mumps vaccine strains and corresponding genotypes.

Mumps vaccine strain	Genotype	Manufacturer
Jeryl-Lynn	A	Merck/Aventis Pasteur NIV Sevapharma
Urabe Am9	B	Sanofi GSK Biken
Hoshino	B	Kitasato Institute
Leningrad-4	N	Moscow Bacterial Medicine Institute
L-Zagreb	N	Institute Immunology Zagreb Serum Institute of India
Miyahara	B	Chemo-Sero Ther Research Institute
Torii	B	Takeda Chemicals
NK M-46	-	Chiba
RS (S-12)	H	Razi State Serum and Vaccine Institute Berna Biotech
RIT 4385	A	GSK
S_79_	A	Dalian Jinjang-Andi Bioproducts
Rubini	A	Swiss Serum Institute

Manufacturers’ list is adapted from the publication Kaaijk et al. (2008).

The mumps strains most commonly used in the MMR and MMRV vaccines are described below.

### The Jeryl-Lynn Strain

The Jeryl-Lynn (JL) strain was named after Jeryl-Lynn Hillman, the child from whom the virus was isolated (Buynak and Hilleman, 1966). The strain was initially cultured in embryonated eggs before subsequent culturing in chick embryos. The JL strain was the first licensed mumps vaccine to be used worldwide. This strain has a good safety profile and is not correlated with meningitis when administered either as a monovalent mumps vaccine or with the trivalent MMR vaccine (Hilleman et al., 1968; Peltola and Heinonen, 1986; Ehrengut, 1989; Nalin, 1989). The JL strain belongs to genotype A. Two different JL lineages, 2 and 5, are used to manufacture mumps vaccines in the United States and Europe.

### RIT 4385

The RIT 4385 strain is derived from the JL strain. Its seroconversion characteristics are similar to the JL strain (Usonis et al., 1998; Gatchalian et al., 1999).

### The Leningrad Strain

The Leningrad-3 (L-3) strain was developed in the Soviet Union in the 1960s, and has been used in Russia to produce mumps vaccines since 1981 (Medynicin, 2003). Initially, it was cultured using guinea pig kidney cells and later with Japanese quail embryos. The L-3 strain can be transmitted horizontally, resulting in asymptomatic infection. Additionally, the L-3 strain has been associated with aseptic meningitis.

### The Leningrad-Zagreb Strain

The Leningrad-Zagreb (L-Z) strain was developed in Zagreb, Croatia, by attenuation of the L-3 strain. The L-Z strain is cultured in chick embryo fibroblast cells. Similar to the L-3 strain, L-Z is also associated with risk of aseptic meningitis.

### The Rubini Strain

The Rubini strain, named for the Swiss child from whom it was isolated, was first licensed in Switzerland in 1985. This strain is grown in diploid human cells and belongs to genotype A (Glück et al., 1986). Because many individuals vaccinated with the Rubini strain subsequently contracted mumps infections during outbreaks in Switzerland, Portugal, Italy, and Singapore, the Rubini strain is no longer used for vaccine production (Germann, et al., 1996; Goncalves et al., 1998; Ong et al., 2005).

### Urabe

The highly immunogenic Urabe strain is a genotype B mumps virus that is licensed for vaccine production in Japan (Vesikari et al., 1983). Two doses of MMR vaccine containing the Urabe strain are five times more protective against mumps infections than a single dose. However, cases of meningitis in children vaccinated with the Urabe strain have been reported. The Urabe strain consists of two strains, one of which has been shown to be associated with meningitis (Brown et al., 1991, 1996). Production of mumps vaccines using the Urabe strain was discontinued in 1993.

## Limitations of Currently Licensed Mumps Vaccines

The mumps vaccines currently licensed for use have two limitations: safety and efficacy.

Following administration of the MMR vaccine, transient, local reactions, such pain and tenderness at the site of infection, are typically observed within 2–3 days of inoculation. Systemic reactions presenting as fever (>103°F/39.4°C) can occur 7–12 days after receiving the vaccine in 5–15% of individuals. While vaccination with the JL strain is associated with mild side effects, safety issues related to other vaccine strains remain a concern. For example, the Urabe vaccine strain has been associated with aseptic meningitis; in 1991, 49 cases of aseptic meningitis were observed in children in Japan (Sugiura and Yamada, 1991), whereas nine cases of aseptic meningitis (per 100,000 doses) were observed in the UK (Miller et al., 1993, 2007). The Urabe strain was later removed from vaccines used in Japan and the UK (World Health Organization, 2014). Further, the L-3 strain is casually associated with aseptic meningitis. In one study, 20–100 cases of aseptic meningitis were observed per 100,000 cases (Cizman et al., 1989). The L-Z strain has also been associated with aseptic meningitis following a vaccination campaign (1.4–4.2 cases per 100,000 doses; Dourado et al., 2000). Remarkably, a study by Klein et al. (2010) determined that children aged 12–23 months who received the MMRV vaccine are at increased risk of febrile seizures at 7–10 days after receiving their first dose (1 every 2,300 doses or 4.3 per 10,000 doses) compared with children who received the vaccine as two separate MMR and varicella vaccines.

The low efficacy of mumps vaccines results from an unacceptable drop in the immune response over time, requiring re-immunization to produce a sufficient immune response to protect against infection. Despite the long existence and routine use of mumps vaccines, outbreaks are on the rise. Even countries with high vaccine coverage, such as the United States, Canada, Australia, and some European countries, have manifested massive mumps outbreaks. The re-emergence of mumps outbreaks can be due to primary or secondary vaccine failure. Primary vaccine failure results from an inefficient immune response following vaccination. It is unlikely that the recent mumps outbreaks have been caused by primary vaccine failure. Secondary vaccine failure is the result of waning immunity over time. Many of the individuals infected in recent mumps outbreaks received their last vaccine dose at least 10 years prior to contracting mumps, indicating secondary vaccine failure as the likely cause for the re-emergence of mumps. Importantly, although mumps-neutralizing antibodies are used to detect immunity in previously vaccinated individuals, the threshold titer of neutralizing antibodies required to confer immunity has not yet been established.

The administration of a third dose MMR vaccine to elevate the mumps-specific antibody response is a potential strategy to better control and prevents mumps outbreaks. Starting in 2017, the Advisory Committee on Immunization Practices (ACIP) recommended a third dose of the MMR vaccine for individuals at risk of contracting mumps. A large study investigated the effectiveness of a third dose of the MMR vaccine in more than 20,000 university students and found that participants who received a third dose of the vaccine were 78% less likely to contract mumps compared to those that received only two doses (Cardemil et al., 2017). The study also revealed that the risk of contracting mumps increased after 13 years from the time of the last vaccination. These findings suggest that the administration of a third dose of the MMR vaccine may help prevent mumps outbreaks.

## Future Prospects for Mumps Vaccines

To improve the safety and efficacy of mumps vaccines, improvements are needed in three areas: mumps strain selection for vaccine production, virus propagation techniques, and modern vaccine platforms.

### Selection of Vaccine Strains

All mumps strains used in currently licensed MMR vaccines originated from strains that circulated in the mid-20th century. Most of those strains are genotype A or B, which were the predominant strains circulating in the pre-vaccination era. JL, which is genotype A, is the most commonly used vaccine strain. The Urabe AM 9 and L-Zagreb vaccine strains are genotype B and N, respectively. With the re-emergence of mumps, most currently circulating mumps strains are non-genotype A. Further, phylogenetic studies have shown that the new strains are distinct from those that circulated previously.

Currently, mumps genotype G is predominantly circulating in Europe, United States, Australia, and New Zealand, while genotype C is primarily circulating in India. Mumps genotype F is circulating in China but has been also found in 10 other countries across North America, Europe, and Asia (Cui et al., 2017). An evolutionary study by Cui et al. (2014, 2017) demonstrated that HN and F produce four different protein lineages in F genotype mumps. Although the evolutionary rate for SH is faster, HN and F are more evolutionary informative compared to SH.

The failure of recent studies to detect wild-type genotype A mumps viruses in regions where individuals are immunized with genotype A vaccines may result from vaccine-based selection pressure on the virus, leading to increased heterogeneity and the emergence of genotypes with increased virulence. Additionally, there is growing evidence that the antibody titers required for cross-resistance varies among mumps genotypes. Therefore, it will be crucial to perform further cross-neutralization studies with mumps genotypes currently in circulation. Furthermore, it is crucial to conduct studies of genotype-specific responses may impart stronger general immunity in specific regions depending on the genotypes circulating within each region. It is essential to monitor the strains that are currently in circulation to determine which strains are predominate in different regions, and thereby improving the selection of vaccine strains for use in certain populations or regions. A limited number of studies have investigated the antigenic relationship between different mumps genotypes and strains. One such study determined that cross-neutralization varies within genotype A mumps strains. Specifically, the neutralization activity of mumps strains JL, SBL-1, and Kilham, were determined to be distinct and represent individual serotypes of genotype A.

### Improved Propagation by Tissue Culture

The MMR vaccine is produced from chicken embryo fibroblasts. It is essential to produce a vaccine without serial passaging in host cells to avoid adaptation-induced antigenic drift of virus strains. Mumps strains grown in human tissue culture would retain the immunogenic epitopes of the passaged virus. For example, the use of human embryonic kidney cells, such as HEK 293 cells, might ultimately lead to an improved mumps vaccine.

### Novel Vaccine Platforms

Development of improved mumps vaccines has progressed slowly. Modern vaccine platforms, such as those based on viral subunits, plasmid DNA, mRNA, or self-amplifying mRNA, should be explored for applicability to mumps vaccine development. Platforms based on DNA and mRNA are rapid, scalable, and do not require propagation of the virus. In addition, it is possible to select the specific immunogen needed to elicit the appropriate immune response. In mumps, the HN protein is responsible for stimulating the neutralizing immune response. Some progress has been made with subunit vaccines, recombinant mumps vaccines, and DNA vaccines.

Liang et al. (2008) described a subunit vaccine consisting of a purified mumps and HN antigen obtained using the mumps SP strain. IRC mice and rhesus monkeys were vaccinated with 10 or 20 μg antigen with an aluminum hydroxide adjuvant. A booster dose was administered 4 weeks later. The HN antigen was immunogenic in animals and induced specific HN neutralization activity. Further, 4 weeks after receiving the booster dose, rhesus monkeys were challenged and found to have complete protection from the wild-type mumps virus. Biao He and colleagues (Xu et al., 2014) created genetically modified mumps strains using a clinically isolated mumps virus. The recombinant strains lacked the SH gene (rMuVΔSH), the V gene (rMuVΔV), or both the V and SH genes (rMuVΔSHΔV). Deletion of the V or SH genes was previously shown to reduce mumps virus neurotoxicity (Xu et al., 2011). The genetically modified mumps strains were administered as potential vaccines intranasally or intramuscularly in BALB/c mice. The rMuVΔSHΔV strain elicited neutralizing antibody responses comparable to those elicited by either the rMuVΔSH strain or the rMuVΔV strain, and stronger than those elicited by the JL strain. In addition, the rMuVΔSHΔV strain elicited a T cell response (Xu et al., 2014).

A DNA vaccination platform was used to design a full-length complementary DNA (cDNA) clone of recombinant MuV-S79 mumps virus by reverse genetics (Zhou et al., 2019). The cDNA clone was administered intranasally to cotton rats to test its efficacy as a potential vaccine. The experimental vaccine elicited an efficient, long-lasting neutralizing antibody immune response, as evidenced by sera obtained and tested 4 weeks after immunization. The vaccine induced complete protection against challenge with a wild-type mumps strain (Zhou et al., 2019).

## Conclusion and Future Prospects

Although widespread use of the two-dose MMR vaccine has been largely successful, mumps outbreaks have been on the rise since the 1990s. Overall, only minor progress has been made to improve mumps vaccines due to a number of unresolved challenges facing researchers. These include limited studies regarding mumps vaccine-induced immunity and the lack of cross immunoprotection between mumps genotypes and strains.

One explanation for the re-emergence of mumps cases is secondary vaccine failure. Many individuals contracted mumps infections at least 10 years after receiving their last vaccination. To better understand secondary vaccine failure, it is vital to determine neutralization titer (NT) levels of the HN neutralizing antibodies required to prevent new mumps infections. Further, determining the half-life of IgG antibodies will be crucial. Determining whether adding a third booster dose to the standard two-dose vaccine series would enhance immunity and produce a higher titer of antibodies against conserved epitopes between various mumps strains would also be a worthy endeavor.

Another explanation for the re-emergence of mumps is antigenic variation; new strains evade the protective neutralizing immunity elicited by the vaccination. While mumps is believed to be serologically monotypic, few molecular surveillance studies of mumps genotypes and strains suggest the existence of antigenic escapes. Therefore, similar to work research on other emerging and re-emerging viruses, large-scale immuno-informatics analyses (Lohia and Baranwal, 2014; Almansour and Alhagri, 2019) are essential for the identification of mutant mumps strains that can be further assessed for potential antigenic mutations. Virus bioinformatics and immunoinformatics resources have provided an enormous body of data to facilitate the study of genetic and antigenic variation in viruses. For example, MMR viruses database (MMRdb) is a comprehensive bioinformatics database containing genomic and protein sequences for the MMR viruses (Almansour et al., 2018). For example, MMRdb can assist researchers in tracking the temporal and geographic spread of mumps and to monitor sequence heterogeneity and antigenic diversity across mumps strains. Interestingly, Abrams et al. (2014) presented a method for predicting regions of high outbreaks potential in Belgium using informed mumps serological survey data and mumps vaccination coverage information. Likewise, Béraud et al. (2018) applied similar approach to explore and map MMR resurgence in France using inferred servo-prevalence cross-sectional studies and vaccination coverage data.

In the future, it may be possible to develop genotype-specific mumps vaccines targeting emerging strains in a region-specific manner. Development of a polyvalent vaccine combining diverse genotypes and strains in circulation may provide another solution to improve mumps vaccines and increase immunogenic cross-neutralization.

In summary, further research is needed to control and prevent future mumps outbreaks. Collaborative, interdisciplinary research efforts by virologists, immunologists, vaccinologists, and virus bioinformaticians, are necessary to monitor and evaluate genetic and antigenic diversity of mumps viruses. Identification of conserved versus variable HN epitopes may be critical to identify mutant mumps genotypes and strains that evade immune responses. Additionally, utilizing a computer-aided vaccine design approach that includes multiple cross-neutralizing epitopes could potentially enhance vaccine coverage and mumps strains cross-protection.

## Author Contributions

The author confirms being the sole contributor of this work and has approved it for publication.

### Conflict of Interest

The author declares that the research was conducted in the absence of any commercial or financial relationships that could be construed as a potential conflict of interest.
